# The Effect of Nonthermal Pretreatment on the Drying Kinetics and Quality of Black Garlic

**DOI:** 10.3390/molecules28030962

**Published:** 2023-01-18

**Authors:** Klaudia Masztalerz, Tomasz Dróżdż, Paulina Nowicka, Aneta Wojdyło, Paweł Kiełbasa, Krzysztof Lech

**Affiliations:** 1 Institute of Agricultural Engineering, The Faculty of Life Sciences and Technology, Wrocław University of Environmental and Life Sciences, Chełmońskiego 37, 51-630 Wroclaw, Poland; 2 Faculty of Production and Power Engineering, University of Agriculture in Krakow, Balicka 116 b, 30-149 Krakow, Poland; 3 Department of Fruit, Vegetable and Nutraceutical Plant Technology, Wrocław University of Environmental and Life Sciences, 50-375 Wroclaw, Poland

**Keywords:** antidiabetic potential, antioxidant capacity, constant electric field, drying, magnetic field, pretreatment, pulsed electric field, total phenolic content

## Abstract

Black garlic is obtained from regular garlic (*Allium sativum* L.) through the aging process and consequently gains many health-promoting properties, including antidiabetic and antioxidant. However, the material is still prone to microbiological deterioration and requires a long time to dry due to its properties. Therefore, this study aimed to investigate the effect of various drying methods on the quality of black garlic as well as determine the influence of selected nonthermal pretreatments on the drying kinetics and quality of black garlic, which is especially important in the case of the materials that are difficult to dry. The Weibull model was chosen to describe drying kinetics. Additionally, color, water activity together with antioxidant activity, phenolic compounds, and antidiabetic potential were determined. This study found that the application of a pulsed electric field (PEF), a constant electric field (CEF) as well as a magnetic field (MF) significantly reduced the time of drying (by 32, 40, and 24 min for a PEF, a CEF, and a MF, respectively, compared to combined drying without the pretreatment), and resulted in high antidiabetic potential. However, the highest content of phenolic compounds (1123.54 and 1125.36 mg/100 g dm for VMD125 and CD3h-VMD, respectively) and antioxidant capacity (ABTS = 6.05 and 5.06 mmol Trolox/100 g dm for VMD500 and CD6h-VMD, respectively) were reported for black garlic treated by vacuum-microwave drying and combined convective pre-drying followed by vacuum-microwave drying. Overall, the nonthermal pretreatment decreased the time of drying and showed very good efficiency in maintaining the antidiabetic potential of black garlic, especially in the case of the materials pretreated by a constant electric field (IC_50_ = 99 and 56 mg/mL, for α-amylase and α-glucosidase, respectively).

## 1. Introduction

Black garlic is produced during the aging of fresh garlic (*Allium sativum* L.). The process is usually performed for an extended period of time (up to several weeks) at increased temperature (60–90 °C) and high relative humidity (50–95%) [1]. Consequently, the material changes its physical and chemical properties, acquiring the typical dark color in the process. The main bioactive compound responsible for the properties of black garlic is S-allyl cysteine (SAC), which is formed as a result of the conversion of unstable alliin present in fresh garlic [2]. Moreover, the strong taste and smell of garlic changes due to the decreased amount of allicin, which is responsible for its strong off-flavor [3]. An increasing amount of Maillard reaction products in black garlic also influences the taste and aroma, which results in a typical sweet and sour taste that some describe as plum [4]. Black garlic can be characterized by many health-promoting properties, including anti-inflammatory [5,6], antidiabetic [7,8,9], anticarcinogenic [10,11] as well as the ability to reduce blood pressure [12,13]. These properties make black garlic a very attractive functional food ingredient that can be used in the development of various snacks or other food products, especially in Japan, China, Korea, and also in the USA, where it is gaining recognition [14]. Moreover, fresh garlic is already recognized and accepted in society and there is already a big market for garlic-derived products [1]. Hence, black garlic is among the fastest-growing health food [15]. Therefore, application of various treatments, including drying, can enable obtaining powders that could be used as spices or additives to functional food products.

Convective drying is among the most common drying methods. The process is based on the principle of water evaporation as a result of airflow through the material that absorbs the moisture from the surface. This is followed by the internal diffusion of moisture from the inside of the sample to the outer layer to enable water evaporation. Convective drying has previously been used in studies on garlic [16], Thai basil [17], hemp flowers [18], and kiwiberry [19]. On the other hand, vacuum and vacuum-microwave drying enable reaching a lower final moisture content in the final product. Moreover, vacuum-microwave drying can significantly accelerate the drying process due to volumetric heating occurring as a result of microwaves application, which is intensified by the pressure diffusion mechanism of the Darcy type, resulting from the pressure gradient between the center of the material and surrounding vacuum. These methods were previously used, among others, in studies on true lavender [20] and *Cassia alata* [21]. Drying is a very energy-intensive process, which is mainly due to the long processing times. This can be reduced by combining different drying methods, such as combined convective pre-drying to remove easily accessible unbound water followed by vacuum-microwave finishing drying aimed at reducing the drying time and improving the quality of the material. As a result, significant energy savings can be observed, such as in studies on garlic [16] as well as osmotic dehydration and drying of apples [22].

Another way of reducing process duration and improving the quality of dried products is the application of various pretreatment methods. The most common are thermal methods, including freezing, blanching, or osmotic dehydration. However, nonthermal methods are also gaining recognition due to their effect on the shortening of the drying duration and limiting microbiological contamination [23]. Among these methods is a pulsed electric field (PEF). The use of a PEF is a pretreatment method based on the application of a very short, high-intensity electric field that leads to a cell membrane disintegration [24]. Consequently, electroporation occurs and leads to the intensification of internal water diffusion and therefore a significant reduction in drying time as shown in recent studies on parsnips [25], onions [26], carrots [27] and red bell pepper [28]. Moreover, the application of a PEF as a pretreatment did not affect the nutritional value of apple juice but led to an inactivation of microorganisms extending the product shelf life [29] as well as improving the microbiological safety of the product. A PEF was also previously used before osmotic dehydration, where it showed a significant reduction in the time of osmotic dehydration of blueberries [30]. Another type of nonthermal pretreatment is a constant electric field (CEF). In this method, the material is placed between the electrodes, and a generated constant electric field interacts with the material changing its properties. A CEF was previously applied in studies on Camellia [31] and *Cannabis sativa* [32]. A magnetic field (MF) has been used to accelerate the germination and growth of various seeds, including sunflower seeds [33] as well as in the freezing of plant materials due to its positive effect on the formation of small ice crystals and enhanced freezing rate [34,35]. Moreover, this method was used during convective drying in order to change its properties and accelerate water removal during drying [36].

Application of various nonthermal pretreatment methods such as a pulsed electric field, a constant electric field, and a magnetic field before drying has previously been discussed in the literature, but never in terms of black garlic drying. Therefore, this study aims to determine the influence of process parameters and nonthermal pretreatments on the drying kinetics and quality of the final product, including changes in the antioxidant and antidiabetic potential of black garlic after treatment.

## 2. Results and Discussion

### 2.1. Physical Properties of Black Garlic

Table 1 shows the physical properties of black garlic after thermal and nonthermal treatments. In the course of nonthermal pretreatment, the moisture content of the material changed according to the used method, namely, after PEF + H_2_O, the moisture content was equal to 1.53 kg/kg; during CEF + H_2_O, *Mc* = 1.21 kg/kg; and for MF + H_2_O, *Mc* = 1.51 kg/kg. On the other hand, while applying a constant electric field and a magnetic field without water, the samples lost some moisture during pretreatment, resulting in *Mc* = 0.49 and 0.50 kg/kg for a CEF and a MF, accordingly, which is significantly lower compared to fresh material (*Mc* = 0.66 kg/kg). This is due to the effect of nonthermal pretreatment, which, when performed with the material immersed in water, increased the *Mc* as a result of water absorption [37]. The studies by Rizvi Alam et al. [25] and Rahaman et al. [38] also showed that the addition of water when using a PEF, a CEF, and a MF is crucial to ensure the uniform distribution of an electric field in the chamber.

The changes in the moisture content as a result of nonthermal pretreatment did not negatively affect the final moisture content after drying (Table 1). When considering the final *Mc* of the material after drying, it can be seen that a higher power of magnetrons during VMD facilitated water removal and enabled obtaining among the lowest values of the *Mc*. Similar results were reported in this study on vacuum-microwave drying of garlic [39]. Samples treated by a PEF, a CEF, or a MF and then combined drying reached a lower moisture content in general. The nonthermal treatment changed the properties of the material and destroyed the cell structure, which led to higher water loss during the process. However, when considering the water activity, it can be seen that even though the pretreated samples exhibited a lower *Mc*, the water activity was higher compared to in non-pretreated samples. This can also be explained by the effect of the pretreatment. When a PEF is applied to the material, electroporation occurs and destroys the cell structure, which not only facilitates water removal but also releases water and chemical compounds from the material matrix [38,40,41]. Consequently, water is more accessible, which leads to increased water activity, even though the material has a lower moisture content than non-pretreated samples. This is in line with the findings presented by Nowacka et al. [24]. Nonetheless, the main factor responsible for microbiological safety is water activity, which in each variant is still below 0.6, making it relatively stable and safe as no proliferation occurs at these values of aw [42].

**Figure 1:**
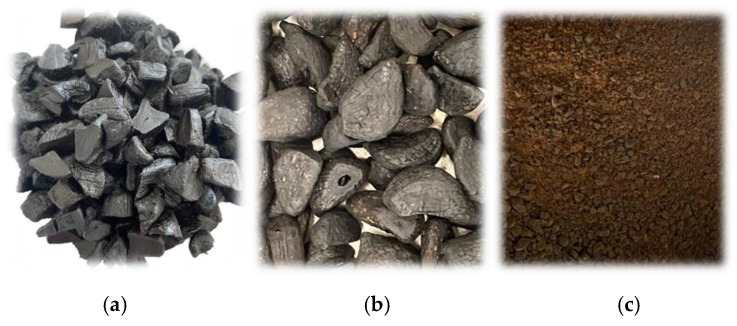
Fresh black garlic sample (**a**), black garlic after drying (**b**), and black garlic powder (**c**).

Color is among the most important characteristics from the consumer’s point of view. Black garlic is characterized by a very intense dark brown or even black color as a result of the formation of melanoidins in the material during the aging of fresh garlic [15]. Color analysis showed that the darkest samples (with the lowest L*) were the ones where vacuum drying was used as a finishing drying method (CD60-9h/VD60 and CD70-9h/VD60). Thus is due to the lowest temperature of the sample when this method was applied (below 60 °C and below 70 °C, respectively). While other drying methods were used, an increase in the L* parameter could be reported which can be explained by the thermal effect in the material. As a result of drying and temperatures reached during the process, the black garlic samples turned brown instead of the very intense dark color of the fresh material, which could be noticed as increased L* and *BI* parameters (Figure 1). Despite the highest temperature obtained when the material was dried by VMD500, the *BI* is the lowest among all VMD samples. This can be explained by the exposure time of the material to the high temperature which was the shortest when dried at 500 W (VMD500). Comparison of the samples pretreated by nonthermal methods and untreated samples dried by CD70-3h/125W showed that the pretreatment led to a decrease in L* after drying which can be explained by the shorter time of vacuum-microwave finishing drying and consequently shorter time of thermal treatment. To the best of the authors’ knowledge, there are no studies regarding the color changes during the processing of black garlic, which can be affected by thermal treatment as shown in this study. Therefore, future studies on this issue are needed.

### 2.2. Drying Kinetics

**Figure 2:**
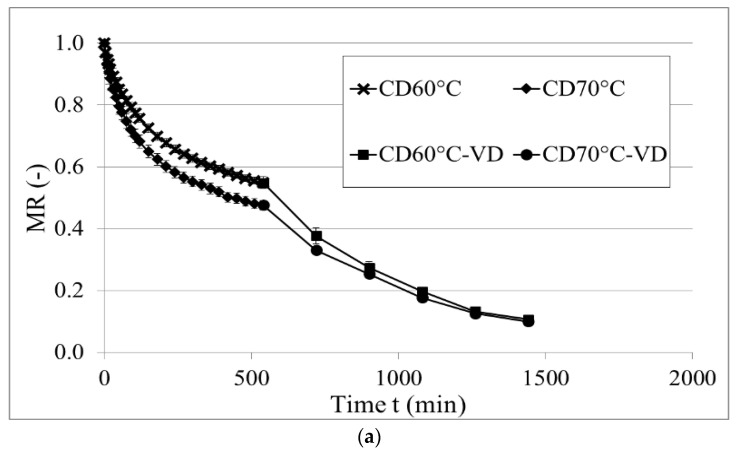

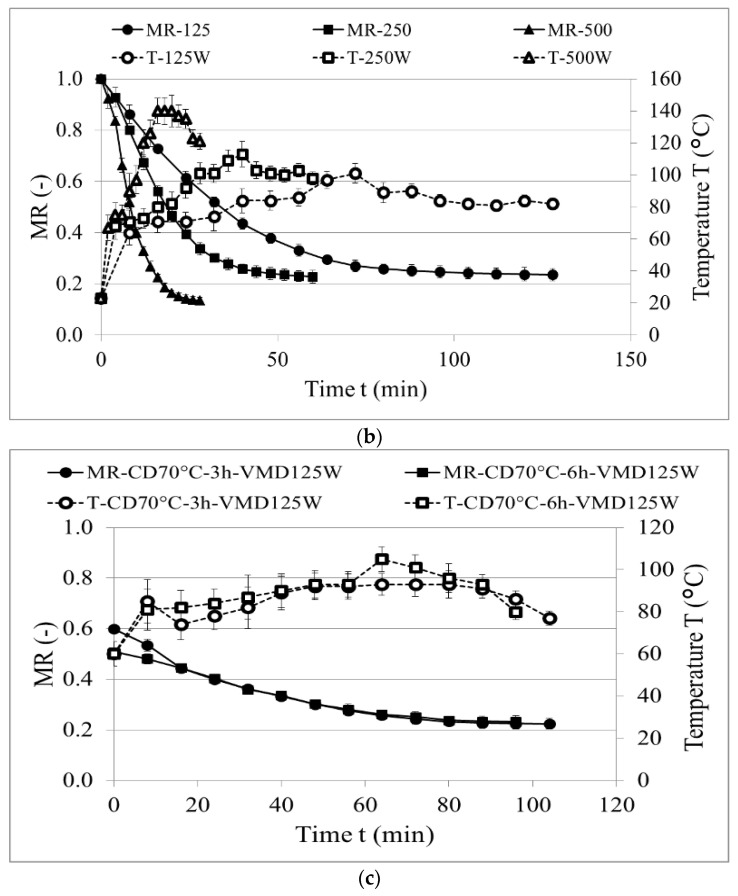
Drying kinetics of black garlic dried using convective pre-drying and vacuum finishing drying (**a**), vacuum-microwave drying (**b**), and combined convective pre-drying followed by vacuum-microwave finishing drying (**c**).

Figure 2 shows the drying kinetics of black garlic treated by convective pre-drying followed by vacuum finishing drying, vacuum-microwave drying, and combined method consisting of convective pre-drying and vacuum-microwave finishing drying. As can be seen, the higher temperature of hot air during convective drying resulted in faster water evaporation and lower MR after 540 min of drying [43]. Then, the application of vacuum drying enabled reaching a lower moisture content while limiting the negative effect of temperature and oxygen, which is typical during CD [44]. While considering the drying kinetics during vacuum-microwave drying, it can be seen that application of VMD reduced the time of drying by 91% in the case of VMD125 compared to CD-VD. Moreover, the higher power of magnetrons during drying resulted in more intense evaporation and further reduced the drying time from 128 at 125 W to 28 at 500 W. This is consistent with previous studies on sour cherries [45] and pears [46]. This can be explained by volumetric heating and temperatures generated during drying. As can be seen, the application of higher power increased the surface temperature of the material up to 140 °C, while drying at 125 W maintained temperatures below 100 °C. Similar behavior was reported by Figiel and Callin-Sanchez et al. [16,39]; however, the temperatures obtained were lower than reported here. This is due to the structure changes occurring in the aging of garlic. As a result, black garlic is much softer, with a gelatin-like texture that disrupts water evaporation and leads to heating up of the material during VMD [47].

According to Figure 2c, it can be seen that increasing the time of convective pre-drying from 3 to 6 h did not affect the drying time during vacuum-microwave finishing drying. Another 3 h of the CD only shortened the VMD time by 8 min. Therefore, prolonged CD resulted in only a little lower MR and this effect was only significant during the first two cycles of VMD. Afterwards, a similar MR was reached and maintained throughout the drying process, which shows that 3 h of convective pre-drying is enough and further prolonging the process does not bring any substantial gains. Different results were obtained in a study on garlic, where a longer time of convective pre-drying resulted in a significant reduction in vacuum-microwave finishing drying duration [16]. However, the proposed pre-treatment times were shorter than the ones presented in this study. Similarly, the study by Castillo-Girones et al. showed that the earlier the switch to vacuum-microwave drying the shorter the overall drying time [48]. This is due to the absorption of microwaves by the water dipoles that are located in the whole volume of the material. Consequently, a higher drying rate can be observed and more intense evaporation occurs compared to convective drying. However, the intense evaporation at the beginning of VMD can exceed the capacity of the vacuum pump [49]. Therefore, it is important to carefully select the process parameters and future research is needed to further optimize the combined drying parameters in black garlic drying.

Based on this, convective pre-drying at 70 °C for 3 h followed by vacuum-microwave finishing drying at 125 W was selected as an optimal method for black garlic drying in the first stage of the experiment. These parameters were then selected for the drying of the materials pretreated by nonthermal methods, i.e., a pulsed electric field, a constant electric field, and a magnetic field.

**Figure 3:**
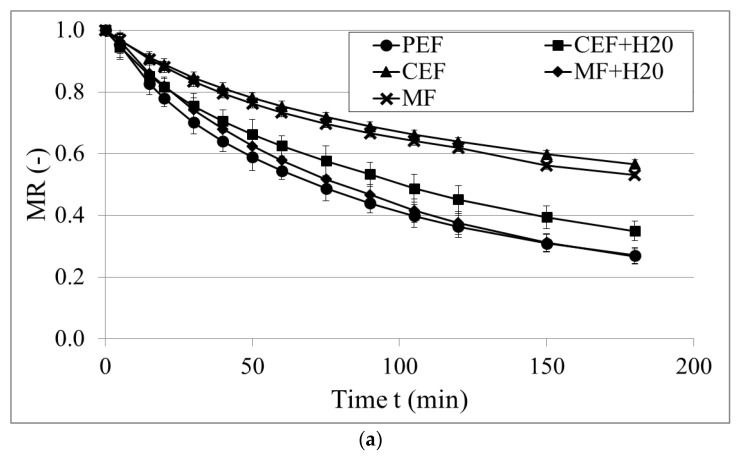

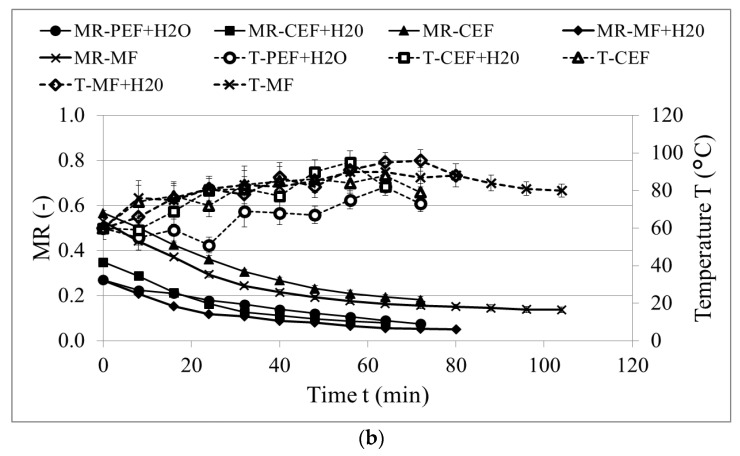
Drying kinetics of pretreated black garlic using a pulsed electric field (PEF), a constant electric field (CEF) and a magnetic field (MF) followed by drying using convective pre-drying (**a**) and vacuum-microwave finishing drying (**b**).

Figure 3 shows the drying kinetics of the samples pretreated by a pulsed electric field, a constant electric field, and a magnetic field and then dried by convective pre-drying and vacuum-microwave finishing drying. As discussed before, nonthermal pretreatment was performed in water, which changed the initial moisture content in the material. Therefore, the MR was applied to compare the drying kinetics of the materials with a different initial *Mc*. Since the samples pretreated in water had a higher *Mc, a* more accelerated reduction in the MR in the course of drying could be observed. Overall, samples pretreated in water showed a lower final MR than the samples pretreated directly, without water. Water provided the necessary conditions for the uniform distribution of electric fields, which led to a more effective influence of a PEF, a CEF, and a MF [38]. Moreover, PEF pretreatment changed the properties of the material to the highest extent which resulted in the lowest MR after CD and rapid removal of water during drying. This is due to the effect of a PEF, which destroyed the cell structure and consequently allowed for more intense water evaporation during convective drying, which is consistent with the studies on onions [26,37]. Among the lowest MR values was obtained for the samples pretreated by MF + H_2_O. This can be explained by the effect of the magnetic field that affects the porosity of the material and as a result, considerably shortens the drying time such as in the studies by Memmedov et al. [36]. A CEF and CEF + H_2_O showed the highest MR in those two groups (processed with/without water). This is due to the relatively mild effect of a constant electric field on the material compared to a PEF.

Several mathematical models were used to describe the experimental data for drying kinetics in this study, and among them, the Weibull model was selected to be applied in this study (Equation (1)).

(1) $$MR=a-b\cdot e^{-k\cdot t^{n}}$$

It can be seen that in all drying variants, R^2^ was above 0.99 and RMSE below 0.01 which shows a very good fit (Table 2). This model was previously used to model drying kinetics in figs [50], lemongrass [51], quince [52], and sultana grape fruits [53] and, in each study, showed a very good fit and ability to accurately describe the experimental data. Based on the parameters presented in this study, it can be seen that the drying constant represented as the *k* parameter increased when the power of magnetrons was higher during VMD. Similarly, *k* values were higher when a higher temperature during convective pre-drying was applied.

### 2.3. Chemical Analysis

#### Bioactive Compounds

Numerous scientific studies have confirmed that phenolic compounds, known as bioactives, have many therapeutic and preventive properties useful for treating chronic diseases, including obesity, diabetes, inflammatory, and neurological diseases [54,55]. Therefore, the phenolic content in the analyzed black garlic samples is shown in Table 3. In the case of all obtained samples, amongthree3 identified groups of phenolics, the dominant ones were flavan-3-ols (monomers and dimers), followed by polymeric procyanidins, and phenolic acids. That consisted of, on average, 96%, 3%, and 1% of all identified compounds, in the case of samples in which the drying process was performed, and, respectively, 90%, 8%, and 2% in fresh black garlic (without additional processing). A significantly higher content of all analyzed phenolics was indicated for black garlic dried by VMD methods (125, 250, and 500 W) and also combined CD-VMD methods (70 °C + 125 W), which were, on average, 2-fold higher when compared with fresh black garlic (Table 3). In turn, the lowest total polyphenols content was determined in samples treated by a PEF. In general, it can be concluded that the drying process resulted in a significant increase in monomeric and dimeric flavan-3-ols, with the simultaneous degradation of polymerized compounds and phenolic acids, while the use of VMD125 W, CD70 °C-3h/125W, and MF + H_2_O did not affect the decrease in phenolic acids.

So far, many studies have been conducted in which the content of polyphenolic compounds in fresh and black garlic was compared. They showed a several-fold higher concentration of bioactive compounds in the processed product compared to fresh garlic [15,56]. To the best of the authors’ knowledge, this is the first time the effect of additional drying on the physicochemical properties of black garlic is being studied. It was shown that it is particularly advantageous in the context of the accumulation of polyphenolic compounds, especially the monomeric and dimeric fractions of flavan-3-ols, to use VMD or combined drying consisting of convective pre-drying and vacuum-microwave finishing drying. This could be due to the heating process improving phenolic content as a result of the cleaving of bound forms (glycosylated, and esterified), thus leading to the increase in free forms of polyphenols. In addition, another reason for an increase in these compounds in the dried sample is the inhibition of enzymatic oxidation involving the antioxidant compounds, including polyphenols. Moreover, an increase in the bioactive compounds could be due to an increase in the levels of polyphenols obtained from the later phase of the browning reaction [56,57].

### 2.4. In Vitro Pro-Health Potency and Antioxidant Capacity

#### Antioxidant Capacity

The analysis of the antioxidant activity of the examined black garlic varieties showed that the type of pretreatment, drying method, and conditions significantly (*p* < 0.05) affected the antioxidant capacity of the tested samples. The antioxidant properties of black garlic varied widely. The highest antioxidant potential measured by the ORAC method was shown by the black garlic dried by VMD500W (7.90 mmol Trolox/100 g dm), while the lowest one by the black garlic obtained through PEF + H_2_O (4.38 mmol Trolox/100 g dm). Similar trends, but slightly lower results, were obtained for the ABTS, and FRAP methods. In these cases, the highest antioxidant potential was recorded for the VMD500W—6.05, and 3.73 mmol Trolox/100 g dm (ABTS, FRAP, respectively), while the lowest value was observed for the PEF + H_2_O process—2.03, and 1.09 mmol Trolox/100 g dm (Table 4).

The conducted study showed that the antioxidant activity positively correlated with the content of bioactive compounds, especially monomers and dimmers, flavan-3-ols, and procyanidin polymers. Other authors confirm this relationship. Their studies indicated that the antioxidant activity depends not only on their amount but also on the structure of the compound and the proportion of individual fractions, i.e., anthocyanins, phenolic acids, flavan-3-ols, and flavonols, in the tested material [58,59].

### 2.5. α-Amylase, and α-Glucosidase Inhibitory Effect

According to the estimates by the World Health Organization (WHO), chronic noncommunicable diseases, including diabetes are currently the main cause of death worldwide. Type 2 diabetes is characterized by hyperglycemia with impaired carbohydrate, lipid, and protein metabolism resulting from defects in insulin secretion, insulin action, or both. A rapid postprandial increase in glycemia is due to starch degradation by pancreatic amylase, followed by the blocking of the resultant glucose by intestinal α-glucosidase. Therefore, it is suggested that the inhibition of these enzymes is an important strategy for the management of type 2 diabetes. In addition, several studies have investigated the antidiabetic potential of black garlic. It has been shown that black garlic exhibited ameliorative action on glycometabolic biomarkers in diabetic rats, as well as the ability to decrease blood glucose, glycated hemoglobin, and markedly increase serum insulin [15]. Thomson et al. [7] showed garlic extract significantly attenuated the elevation of serum triglyceride, and lowered lipid peroxidation in the kidney, and liver tissues. Therefore, in the present study, black garlic after various treatments was tested for its ability to inhibit α-amylase, and α-glucosidase (Table 4).

The IC_50_ values of the black garlic for inhibiting α-amylase activity ranged from 61.73 mg/mL to 229.46 mg/mL, and the inhibition effect was the highest for black garlic dried by VMD500W, while samples with CD70 °C-9h/VD60 exhibited the lowest inhibition effect (Table 4). In turn, the ability to inhibit α-glucosidase ranged from 51.21 mg/mL (CD70 °C-3h/125W) to 231.85 mg/mL in garlic treated by MF + H_2_O. Nevertheless, no recurring pattern was observed in terms of the creation of anti-α-amylase, and anti-α-glucosidase potential by type of fraction, the content of polyphenolic compounds, or drying method. In this case, it is worth emphasizing the high efficiency of black garlic after PEF treatment to inhibit both enzymes, especially since this sample was characterized by both low contents of polyphenolics and low antioxidant activity. This may be due to the higher amount of specific amino acids, which are abundantly described in the literature as molecules with a significant antidiabetic potential [60]. Numerous studies describe the key role of amino acids in shaping the health potential of black garlic [15,57]. The CEF + H_2_O treatment may be an effective tool to increase the availability of amino acids in plant materials and thus result in their greater effectiveness in the area of antidiabetic properties, but confirmation of this statement requires detailed studies, which are planned in the future.

## 3. Materials and Methods

### 3.1. Material

Reagents for antioxidant and biological activity tests (α-amylase, α-glucosidase, starch, 2,2′-azino-bis(3-ethylbenzothiazoline-6-sulfonic acid) diammonium salt, TPTZ, p-nitrophenyl-α-D-glucopyranoside) were purchased from Sigma-Aldrich (Steinheim, Germany). Standards for polyphenols were acquired from Extrasynthese (Lyon Nord, France). Acetonitrile, methanol, and formic acid for ultra-performance liquid chromatography (UPLC; gradient grade) were purchased from Merck (Darmstadt, Germany). Black garlic was produced from regular garlic (*Allium sativum* L.) as a result of the aging process. The material used in this study was obtained from the local producer (“PASZKÓW” Farma Tadeusz Kaczmarczyk, Świdnica, Poland). Garlic slices were sliced in half and each sample consisted of 100 g of material of the initial moisture content *Mc* = 0.66 kg/kg db.

### 3.2. Pretreatment Methods

A pulsed electric field (PEF) treatment was performed in prototype equipment built at the University of Agriculture in Krakow, Poland (model ERTEC-SU1 with Line Parameters Analyzer type AS3 Mini) [29,61]. The material was immersed in water and then placed in a treatment chamber between the electrodes. Each time, the material was treated with 300 impulses with 10 s break between the impulses. The strength of the electric field was fixed at 5 kV/cm.

A constant electric field (CEF) was applied in an apparatus consisting of two flat electrodes placed in a chamber with the material in between [32]. The high voltage pulse generator was set to 9 kV. The treatment consisted of 1272 impulses for 10 s each, with a 5 s break between the impulses. Two variants of CEF treatment were performed: with and without the addition of water to the material.

**Figure 4:**
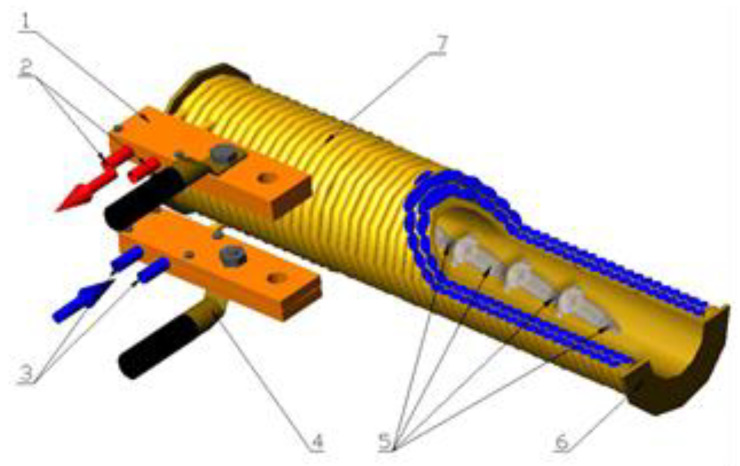
Diagram of the solenoid for magnetic stimulation of black garlic samples: 1—supply chamber; 2—cooling water outlet; 3—cooling water inlet; 4—power cables; 5—sample containers; 6—carcass; 7—coil.

A magnetic field (MF) was used to treat the material alone and with the addition of water using a magnetic field of 100 mT at the frequency of 50 Hz for 2 h using equipment built at the University of Agriculture in Krakow (Kraków, Poland) (Figure 4).

### 3.3. Drying Methods

**Figure 5:**
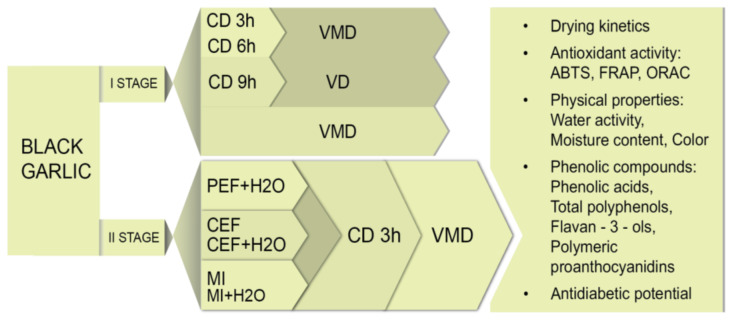
Schematic representation of the two stages of experiments performed in this study and analyses performed in this study. CD—convective drying; VMD—vacuum-microwave drying; VD—vacuum drying; PEF—pulsed electric field; CEF—constant electric field; MF—magnetic field.

In the first stage of the experiment, black garlic cloves were subjected to convective drying (CD) performed using the convective dryer designed and constructed at the Institute of Agricultural Engineering (Wrocław, Poland) [62] at 60 and 70 °C and the air velocity of 0.5 m/s for 9 h (Figure 5). Then, pre-dried black garlic was moved to a vacuum dryer (VD) and dried at 60 °C under 100 Pa (SPT-200, ZEAMiL, Horyzont, Kraków, Poland) until the moisture content of the material was below 13%. Vacuum-microwave drying (VMD) was performed using SM200 dryer (Plazmatronica, Wrocław, Poland) [63] at 125, 250, and 500 W power of magnetrons under the pressure in the range of 50–70 hPa. Combined CD-VMD consisted of 3 h or 6 h of convective pre-drying at 70 °C followed by vacuum-microwave finishing drying at 125 W (CD3h-VMD or CD6h-VMD, respectively).

In the second stage of this study, black garlic pretreated using a PEF, a CEF, and a MF was subjected to convective pre-drying at 70 °C for 3 h and then vacuum-microwave finishing drying at 125 W.

The surface temperature of all the samples was measured using an infrared camera i50 (Flir Systems AB, Stockholm, Sweden). Experiments were carried out in two repetitions.

### 3.4. Physical Properties

#### 3.4.1. The Moisture Content

The moisture content (Mc) of the samples was measured using vacuum-drying (SPT-200, ZEAMiL, Horyzont, Kraków, Poland) at 100 Pa and 80 °C for 48 h. Measurements were performed in duplicate.

#### 3.4.2. Water Activity

The water activity of the samples both before and after treatments was measured using AquaLab Dew Point 4TE (Decagon Devices Inc., Pullman, WA, USA) water activity meter. Water activity was measured at 25 ± 0.5 °C in triplicate.

#### 3.4.3. Color

Color measurement was performed with the black garlic powder obtained after grinding the material using Profi Cook grinder (PC-KSW 1021) after each drying treatment. Then, the sample was placed in a vessel placed on a Chroma Meter CR-400 colorimeter (Minolta Co., Ltd., Osaka, Japan). The color was expressed within CIE L*a*b* color space meaning lightness (L*)*,* hues from red to green according to the values of a* coordinate, and from yellow to blue for b* coordinate. The browning index (*BI*) was calculated based on the equations (Equations (2) and (3)) provided by Subhashree et al. [64]:

(2) $$X=\frac{a^{*}+1.75\cdot L^{*}}{5.645\cdot L^{*}+a-3.012\cdot b^{*}}\;$$

(3) $$BI=\frac{100\cdot \left(X-0.31\right)}{0.17}$$

### 3.5. Chemical Analysis

#### 3.5.1. Determination of Phenolic Compounds, including Polymeric Proanthocyanidins by UPLC

Determination of phenolics in black garlic and black garlic after different treatments was performed as described by Nowicka et al. [65] using an Acquity UPLC system (Waters, Milford, MA, USA) with a photodiode and a fluorescence detector with the mass detector G2 Qtof mass spectrometer (Waters, Manchester, UK). The absorbance values of flavan-3-ols and phenolic acids were read at 280 nm, and 320 nm, respectively. The content of polymeric procyanidins was analyzed by the phloroglucinol method [66]. All samples were measured in triplicate, and the results were expressed as mg per 100 g dry mass.

#### 3.5.2. Analysis of Health-Promoting Properties by In Vitro Methods

To analyze the antioxidant activity, the ORAC (oxygen radical absorbance capacity), FRAP (ferric reducing antioxidant power), and ABTS (2,2′-azino-bis(3-ethylbenzothiazoline-6-sulphonic acid)) methods were used as described earlier by Ou et al. [67], Benzie et al. [68], and Re at al. [69], respectively. The obtained results were presented as mmol Trolox per 100 g dry matter (dm).

The α-amylase and α-glucosidase inhibitory effects (antidiabetic activity) of the black garlic were determined according to the procedure described by Nowicka et al. [70]. In the α-amylase activity determination, the primary sample consisted of black garlic extracts, to which the starch solution, as well as α-amylase solution, were added. The reaction of these components was carried out at 37 °C for 15 min, and then it was stopped with 0.4 M HCl followed by the addition of potassium iodide with iodine.

In the case of α-glucosidase activity analysis, the basic sample including back garlic extracts and enzymes, was incubated at 37 °C for 10 min, then the β-D-glucosidase substrate was added, and incubated as before. Acarbose was included as a positive control for α-amylase and α-glucosidase assay. The absorbance measurement was done at 600 nm and 405 nm for α-amylase and α-glucosidase activity, respectively, using a Synergy H1 spectrophotometer (BioTek, Winooski, VT, USA). Both tests were performed in triplicate, and the results were expressed as IC_50_ values.

### 3.6. Statistical Analysis

Statistica 13.3 software (StatSoft, Krakow, Poland) was used for all statistical analyses. The results were presented as mean ± standard deviation. One-way analysis of variance (ANOVA) was performed in this study. HSD Tukey’s least significance test (*p* < 0.05) was used to determine homogenous groups. Table Curve 2D v. 5.0 (Systat Software, Inc., San Jose, CA, USA) was used to fit mathematical models to the experimental data based on the lowest values of the root mean square error (RMSE) and the highest values of the coefficient of determination (R^2^).

## 4. Conclusions

The impact of convective, vacuum-microwave, and combined drying methods was investigated in this study together with the effect of nonthermal pretreatment including a pulsed electric field, a constant electric field, and a magnetic field on the quality of black garlic. This study found that the application of different pretreatment methods significantly reduced the overall time of combined drying as well as leading to the lowest values of the final moisture content among considered treatment variants. Drying kinetics were described by the Weibull model, which presented a very good fit. Moreover, pulsed electric field and magnetic field treatments proved to be effective in maintaining the health-promoting properties of black garlic, especially in terms of antidiabetic potential. However, vacuum-microwave drying positively affected the phenolic content and antioxidant capacity of black garlic due to the thermal effect that led to the cleaving of bound forms of polyphenols and consequently an increase in bioactive compounds. This study showed that nonthermal pretreatments considerably affect the drying process as well as the quality of dried materials; however, future studies are needed to find the optimal process parameters.

## Figures and Tables

**Table 1:** Water activity (*aw*), moisture content (*Mc*), and color (CIE L*a*b*, browning index—*BI*) of black garlic powders after nonthermal treatments and drying.

Method	*Mc* (%)	*aw* (-)	Color			
			L*(D65)	a*(D65)	b*(D65)	*BI*
VMD 500W	8.16 ± 0.4 ^ab^*	0.2172 ± 0.0054 ^b^	35.35 ± 0.11 ^f^	6.21 ± 0.28 ^a^	8.72 ± 0.68 ^a^	40.8 ± 3.2 ^d^
VMD 250W	13.06 ± 0.63 ^c^	0.229 ± 0.0048 ^b^	38.01 ± 0.08 ^d^	6.9 ± 0.11 ^b^	12.2 ± 0.44 ^f^	51.5 ± 2.0 ^c^
VMD 125W	13.42 ± 0.65 ^c^	0.2891 ± 0.0071 ^a^	37.76 ± 0.17 ^d^	7.71 ± 0.09 ^h^	13.61 ± 0.28 ^g^	59.0 ± 1.6 ^b^
CD70-3h/125W	12.95 ± 0.63 ^c^	0.2936 ± 0.003 ^a^	38.89 ± 0.11 ^e^	8.34 ± 0.09 ^i^	14.8 ± 0.18 ^c^	62.8 ± 1.1 ^ab^
CD70-6h/125W	13.44 ± 0.65 ^c^	0.2994 ± 0.0034 ^a^	39.70 ± 0.30 ^e^	9.06 ± 0.27 ^j^	14.93 ± 0.37 ^c^	63.1 ± 1.5 ^a^
CD60-9h/VD60	8.89 ± 0.43 ^a^	0.3957 ± 0.0058 ^d^	29.08 ± 0.32 ^b^	2.31 ± 0.1 ^d^	2.7 ± 0.12 ^e^	15.3 ± 0.6 ^g^
CD70-9h/VD60	8.57 ± 0.41 ^ab^	0.3479 ± 0.0054 ^c^	29.16 ± 0.48 ^b^	1.66 ± 0.14 ^c^	1.84 ± 0.03 ^d^	10.5 ± 0.5 ^h^
PEF + H_2_O/CD70-3h/125W	10.51 ± 0.51 ^e^	0.4694 ± 0.0133 ^g^	31.00 ± 0.52 ^a^	4.65 ± 0.02 ^g^	4.52 ± 0.1 ^a^	26.3 ± 0.7 ^e^
CEF + H_2_O/CD70-3h/125W	8.89 ± 0.43 ^a^	0.3891 ± 0.0079 ^d^	31.23 ± 0.45 ^a^	4.04 ± 0.04 ^f^	4.53 ± 0.04 ^a^	24.8 ± 0.6 ^ef^
CEF/CD70-3h/125W	8.14 ± 0.39 ^ab^	0.3691 ± 0.0044 ^f^	30.92 ± 0.11 ^a^	3.19 ± 0.05 ^e^	4.26 ± 0.1 ^a^	22.0 ± 0.6 ^f^
MF + H_2_O/CD70-3h/125W	7.26 ± 0.35 ^bd^	0.3485 ± 0.0022 ^c^	33.57 ± 0.18 ^c^	5.9 ± 0.02 ^a^	7.99 ± 0.04 ^b^	39.6 ± 0.4 ^d^
MF/CD70-3h/125W	6.41 ± 0.31 ^d^	0.3211 ± 0.0054 ^e^	34.28 ± 0.1 ^c^	6.72 ± 0.03 ^b^	8.57 ± 0.05 ^b^	42.6 ± 0.3 ^d^

* Values followed by the same letter, within the same column, were not significantly different (*p* > 0.05), according to Tukey’s HSD test.

**Table 2:** Weibull model parameters (*a, b, k,* and *n*) together with the root mean square error (RMSE) and the coefficient of determination (R^2^) according to the used drying and pretreatment method.

Pretreatment	Drying	Constants				Statistics	
		*a*	*b*	*k*	*n*	RMSE	R^2^
-	CD60 °C	0.460	−0.540	0.0180	0.728	0.0014	0.9999
	CD70 °C	0.416	−0.589	0.0290	0.692	0.0033	0.9996
	CD60 °C-VD	−0.001	−0.550	0.0028	0.939	0.0076	0.9959
	CD70 °C-VD	−0.029	−0.506	0.0030	0.901	0.0091	0.9918
	VMD125W	0.230	−0.765	0.0134	1.245	0.0057	0.9994
	VMD250W	0.230	−0.773	0.0136	1.490	0.0023	0.9999
	VMD500W	0.138	−0.864	0.0267	1.630	0.0123	0.9982
	CD70 °C-3h-VMD125W	0.205	−0.395	0.0236	1.060	0.0068	0.9965
	CD70 °C-6h-VMD125W	0.221	−0.287	0.0047	1.450	0.0030	0.9989
PEF + H_2_O	CD70 °C-3h	0.171	−0.837	0.0230	0.866	0.0071	0.9990
CEF + H_2_O	CD70 °C-3h	0.104	−0.900	0.0220	0.786	0.0044	0.9995
CEF	CD70 °C-3h	0.443	−0.559	0.0170	0.858	0.0014	0.9999
MF + H_2_O	CD70 °C-3h	0.139	−0.869	0.0150	0.933	0.0058	0.9993
MF	CD70 °C-3h	0.374	−0.631	0.0180	0.834	0.0052	0.9986
PEF + H_2_O	VMD125W	−0.810	−1.080	0.0075	0.764	0.0037	0.9958
CEF + H_2_O	VMD125W	0.079	−0.272	0.0180	1.300	0.0036	0.9982
CEF	VMD125W	0.0156	−0.410	0.0120	1.270	0.0015	0.9998
MF + H_2_O	VMD125W	0.041	−0.228	0.0460	0.957	0.0054	0.9930
MF	VMD125W	0.139	−0.393	0.0220	1.160	0.0043	0.9986

**Table 3:** The content of phenolic compounds in black garlic after pretreatments and drying.

Method	Phenolic Acids	Flavan-3-ols	Polymeric Proanthocyanidins	Total Polyphenols
	[mg/100 g dm]			
Fresh	11.26 ± 0.01 ^a^*	488.24 ± 2.48 ^i^	43.41 ± 0.23 ^a^	543.92 ± 2.72 ^i^
VMD 500 W	2.19 ± 0.04 ^k^	1088.86 ± 9.32 ^a^	21.25 ± 0.53 ^h^	1112.31 ± 9.86 ^a^
VMD 250 W	1.98 ± 0.03 ^l^	967.67 ± 3.81 ^c^	24.94 ± 0.82 ^f^	994.59 ± 4.66 ^c^
VMD 125 W	10.02 ± 0.38 ^c^	1083.67 ± 15.09 ^a^	29.86 ± 0.05 ^b^	1123.54 ± 15.52 ^a^
CD70 °C-3h/125W	10.14 ± 0.21 ^c^	1086.46 ± 18.13 ^a^	28.76 ± 0.13 ^d^	1125.36 ± 18.47 ^a^
CD70 °C-6h/125W	2.99 ± 0.02 ^i^	1023.53 ± 13.05 ^b^	22.37 ± 0.09 ^g^	1048.89 ± 13.16 ^b^
CD60 °C-9h/VD60	9.46 ± 0.15 ^d^	627.13 ± 9.00 ^g^	17.61 ± 0.25 ^i^	654.20 ± 9.40 ^g^
CD70 °C-9h/VD60	9.07 ± 0.24 ^e^	709.64 ± 4.04 ^f^	24.10 ± 0.03 ^f^	742.81 ± 4.31 ^f^
PEF + H_2_O/CD70-3h/125W	6.55 ± 0.05 ^g^	366.55 ± 4.07 ^j^	29.30 ± 0.09 ^c^	402.40 ± 4.21 ^j^
CEF + H_2_O/CD70-3h/125W	4.09 ± 0.08 ^h^	576.37 ± 2.13 ^h^	11.97 ± 0.19 ^k^	592.42 ± 2.40 ^h^
CEF/CD70-3h/125W	7.54 ± 0.05 ^f^	612.78 ± 9.26 ^g^	25.57 ± 0.15 ^e^	645.89 ± 9.46 ^f^
MF + H_2_O/CD70-3h/125W	10.57 ± 0.24 ^bc^	821.67 ± 10.38 ^d^	25.79 ± 0.15 ^e^	858.04 ± 10.57 ^d^
MF/CD70-3h/125W	2.66 ± 0.01 ^j^	776.50 ± 8.82 ^e^	21.73 ± 0.43 ^h^	800.90 ± 9.26 ^e^

* Values followed by the same letter, within the same column, were not significantly different (*p* > 0.05), according to Tukey’s HSD test.

**Table 4:** Antioxidant, and antidiabetic potential of black garlic dried by different methods.

Method	ABTS	FRAP	ORAC	Inhibition of α-Amylase	Inhibition of α-Glucosidase
	mmol Trolox/100 g dm			IC50 [mg/mL]	
Fresh	2.60 ± 0.24 ^e^*	1.35 ± 0.04 ^f^	6.67 ± 0.03 ^c^	186.56 ^h^	211.24 ^g^
VMD 500 W	6.05 ± 0.31 ^a^	3.73 ± 0.10 ^a^	7.90 ± 0.05 ^a^	61.73 ^a^	63.11 ^c^
VMD 250 W	5.18 ± 0.51 ^b^	3.08 ± 0.04 ^b^	7.85 ± 0.03 ^a^	135.04 ^e^	208.59 ^g^
VMD 125 W	3.50 ± 0.20 ^c^	1.92 ± 0.08 ^d^	6.82 ± 0.03 ^b^	140.87 ^f^	186.71 ^f^
CD70 °C-3h/125W	3.76 ± 0.31 ^c^	2.17 ± 0.06 ^c^	5.50 ± 0.02 ^g^	111.98 ^d^	152.50 ^e^
CD70 °C-6h/125W	5.06 ± 0.16 ^b^	3.07 ± 0.02 ^b^	6.80 ± 0.03 ^b^	83.03 ^b^	51.21 ^a^
CD60 °C-9h/VD60	3.06 ± 0.17 ^d^	1.80 ± 0.07 ^d^	5.51 ± 0.02 ^g^	134.71 ^e^	227.79 ^hi^
CD70 °C-9h/VD60	2.87 ± 0.12 ^de^	1.57 ± 0.02 ^e^	5.89 ± 0.03 ^d^	229.46 ^j^	155.59 ^e^
PEF + H_2_O/CD70-3h/125W	2.03 ± 0.19 ^f^	1.09 ± 0.05 ^g^	4.38 ± 0.03 ^i^	142.86 ^f^	223.77 ^h^
CEF + H_2_O/CD70-3h/125W	2.71 ± 0.19 ^d^	1.18 ± 0.02 ^g^	5.60 ± 0.01 ^f^	99.00 ^c^	56.00 ^b^
CEF/CD70-3h/125W	2.49 ± 0.17 ^e^	1.41 ± 0.05 ^f^	5.18 ± 0.04 ^h^	188.39 ^h^	149.64 ^e^
MF + H_2_O/CD70-3h/125W	2.38 ± 0.23 ^ef^	1.37 ± 0.06 ^f^	5.76 ± 0.02 ^e^	153.09 ^g^	231.85 ^i^
MF/CD70-3h/125W	2.72 ± 0.08 ^e^	1.54 ± 0.02 ^e^	6.75 ± 0.02 ^b^	213.62 ^i^	109.37 ^d^

* Values followed by the same letter, within the same column, were not significantly different (*p* > 0.05), according to Tukey’s HSD test.

## Data Availability

The data that support the findings of this study are available from the corresponding author upon reasonable request.

## Sample Availability

Samples of the compounds are not available from the authors.
